# Passive, Silent and Revolutionary: The ‘Arab Spring’ Revisited

**DOI:** 10.1080/19436149.2016.1177919

**Published:** 2016-05-20

**Authors:** Billie Jeanne Brownlee, Maziyar Ghiabi

**Affiliations:** ^a^ Institute of Arab and Islamic Studies, University of Exeter, 22 Tyndale Road, OX4 1JL, Oxford, UK; ^b^ St Antony’s College, University of Oxford, 62 Woodstock Road, OX1 4JF, Oxford, UK

**Keywords:** *Arab Spring*, *civil society*, Indignados, *Islamic state*, *ISIS*, *Orientalism*, *revolution*, *scholarship of silence*, *social movement*, *counterrevolution*

## Abstract

To counter the trend toward mechanization of research and aridity of critical analysis, this article makes a case for an interdisciplinary quest. To borrow Felix Guattari and Gilles Deleuze’s phrase, we are convinced that ‘everything is political, but every politics is simultaneously a macropolitics and a micropolitics.’ With an eye to open-ended research questions, this article attempts to build a body of theoretical, political and anthropological considerations, which, it is hoped, could function as a case of enquiry into the mechanics of power, revolt and revolution. The objective is to draw comparative and phenomenological lines between the events of the 2011 ‘Arab Spring,’ in its local ecologies of protest, with its global reverberations as materialized in the slogans, acts and ideals of Greek and Spanish Indignados and the UK and US occupy movements. In order to do so, it proposes to clarify terminological ambiguities and to bring into the analytical scenario new subjects, new means and new connections. The article resolves to lay the ground for a scholarship of silence, by which the set of unheard voices, hidden actions and defiant tactics of the ordinary, through extraordinary people, find place in the interpretation of phenomena such as revolts and revolutions.

*What matters most is that people, all men,*

*abandon their sheepish instincts and habits*

*that the millennial slavery inspired them,*

*and they learn to think and act freely.* ^*1*^

The spark for what initially was defined by many as the ‘Arab Spring’ had among its numerous effects that of changing people’s perception about the Arab world.^2^ In the view of most observers, including a majority of people living there, the region was perceived as being in the grip of an authoritarian spell that had slowed down the flow of time and transformed people into docile citizens, unwilling and/or incapable of challenging the *status quo* and their rulers.^3^ The myth of authoritarian survival permeated even more the academic world and policymaking circles, which seemed to have eyes only for those elements that guaranteed stability and longevity to the ruling elite.^4^ The totem of this scholarship was a strong leadership above everything else.

It was not long before the outset of the civil war that Syria was referred to as a ‘successful’ story of authoritarianism, capable of instilling obedience and fear of the ruler into its citizenry, which enabled its metamorphosis into a ‘disciplined society’ of non-thinking subjects.^5^ The idea of a docile citizenry, in political terms, often would concretize in the portrayal of Arab societies as inherently chaotic and unruly.^6^ The 2011 uprising, however, revealed a more complex and vivid reality. The perceptions and understanding of their political lives by people and scholars have changed profoundly, confuting the Western (and Westernized) portrayals of Arab states.

Above all, the misconceptions in much of the pre-2011 scholarship expresses a state of unpreparedness among most of the academic community including ‘us,’ the authors, for assessing the present with critical skills, or in other words *a state of detachedness*. The roots of this cultural and political diagnosis are also likely to be the source of further misconception during this long and conflictual moment of struggle in the Arab world. It permeates, perhaps, in the way we apply the dissecting tools of analysis, our disciplines and our field of enquiry. Indeed, the detachedness of the scholarship from the actual field of inquiry is, *inter alia*, part and parcel of the dominance of today’s methodological approaches. With the shift in the social sciences toward quantitative and macro-data methodologies, *in tandem* with the quasi-monopoly of rational choice theory, one need not to be surprised that the inner-workings and ground-level changes that inevitably feature in a (pre)revolutionary moment, are lost in translations. From numbers to phenomena, if the latter endeavor actually occurs, social science scholars have remained mostly unaware of radicalizing dynamics.

To counter this trend toward mechanization of research and aridity of critical analysis, this article makes a case for (and actually attempts to perform) an interdisciplinary quest. To borrow Felix Guattari and Gilles Deleuze’s phrase, we are convinced that ‘everything is political, but every politics is simultaneously a *macropolitics* and a *micropolitics*.’^7^ With an eye to open-ended research questions, this article attempts to build a body of theoretical, political and anthropological considerations, which, it is hoped, could function as a case of enquiry into the mechanics of power, revolt and revolution. In order to do so, it proposes to clarify terminological ambiguities and brings into the analytical scenario new subjects of enquiry.

This article preconfigures three salient points in framing, dissecting and analyzing the events that go under the label of ‘Arab Spring.’ Firstly, it brings into the discourse the terminology—and related historical contextualization—in approaching the ‘Arab Spring.’ Strongly embedded in Orientalist discourse, terminological preferences are part of a process of defining political phenomena; they bear significance *per se*, apart from revealing aspects of the mindset of the interpreter. Then the article proceeds to contest the Eurocentric linguistic, thematic and methodological *modus operandi*. The objective is to draw comparative and phenomenological lines between the events of the ‘Arab Spring’ in their local ecologies of protest and contention, with the *global* reverberations as materialized in the slogans, acts and idea(l)s of Spanish *Indignados*, Greek protesters, UK and US occupy movements. Eventually, the aim is to lower the perspective and cast light on what lies ‘below’ the surface of Western eyes, defying the taxonomic imperative of much of the Orientalizing analysis.

In doing so, the article resolves to lay the ground for a *scholarship of silence*,^8^ by which the set of unheard voices, hidden actions and defiant tactics of the ordinary, through extraordinary people, find place in the interpretation of phenomena such as revolts and revolutions. The sources and analysis are inspired by the combination of the authors’ distinct researches, which draw on substantial field observation, both on-line and on the ground and participation in events related to the broad political mobilization in the last decade. These elements constitute the backbone of the hermeneutic reflections on which the article is based, but they do not fully materialize in the narrative of the text, which, for reasons of contingency and pertinence, privileges a theoretical grounding of them.

## Terminological Schizophrenia: A Question of Words and Politics

When dealing with political and social events outside the boundaries of Europe and North America, scholars have had the tendency at times to use exogenous categories, derived from the social sciences and contextualized in the development of Western history. The downside of this approach was in being out of touch both with the changing realties of globalized societies and with the local dimensions in Middle Eastern countries. As soon as the events unfolded in Tunis, Cairo and other Arab capitals, for the sake of immediacy, observers brought back from the cold the toolkit of analysis that had been applied to other regions of the world. If the democratic wave had swept from Latin America to Europe and East Asia in the last few decades, skipping the Arab shores,^9^ time had come to reconsider *clinically* the pathology of authoritarianism in its last and, perhaps more entrenched, manifestation.

However, the whole system of paradigms and theories, on which the West stood, crumbled when the popular uprising broke out in Tunisia and unexpectedly developed into a regional phenomenon. Political, academic, and media analysts felt deep incredulity: From where did the uprisings arise? How can one explain and interpret the events? And did this mean that Arab societies and states no longer were inherently authoritarian? The academic world recited a *mea culpa* for having missed the Arab revolts. Gregory Gause modestly admitted that political scientists had been inebriated by the myth of authoritarian stability, failing to capture the ‘forces for change that were bubbling from below, and at times above, the surface of Arab politics.’^10^ The myth of change *of* regimes often passed through change *in* regimes, a development that largely had been unnoticed.^11^

Scholars of Arab affairs and media analysts competed in labelling the 2011 upheavals with a number of symbolic and trendy definitions as the ‘Arab revolutions,’ the ‘Arab Spring,’ the ‘Arab Awakening’ and rather colorfully, if not slightly politically incorrect toward the Japanese, the ‘Arab tsunami.’^12^ As terminology is a locus of lasting influence in the understanding and imagining of historical events, it is surprising how uncontested and undebated has been the adoption of the terminology for the events in the Arab world.^13^ The rush in naming the upheavals resulted in the coining of expressions that have proven piecemeal, incorrect and hasty. We hereby offer an initial dissection of this terminological schizophrenia.

Trying to find definitions for events is like forcing someone to wear tight shoes: They never fit perfectly! In this regard, the late political theorist Fred Halliday suggested that,

Definitions of revolution are, like all definitions in social sciences, conventional: revolutions are not—any more than are nations, classes, even events or date—objectively given ‘things’ waiting to be unearthed or identified like objects of natural science. They are phenomena which human subjects choose to group, on the basis of criteria of significance and recurrence, into one category rather than another.^14^

Yet, the fundamental need to provide analytical categories, surely of the dynamic type, is crucial to the world of a social scientist and, for that matter, a scholar of politics. The use of social theory is as important as the need to contextualize the theory in the localities of the phenomena. Terminology is not chosen randomly or methodologically insignificant. Terminology, *pari passu* with theory, allows the expression of ideas and phenomena beyond the limited field of study.^15^ It generates, hence, a scholarship, which without terminology, would be hard, if not impossible, to configure or even to use as a reference. Language, wrote Walter Benjamin, is not simply and only the communication of what can be communicated, but also it is the symbol of what cannot be communicated.^16^

## Arab Spring, Arab Revolution, Arab Awakening or Arab Uprising?

The Arab uprisings have been defined as ‘spring’ or ‘revolutions’ from the very beginning. The term ‘spring’ is a metaphor, which recalls the brief Czechoslovakian Spring of 1968, quickly halted by the Red Army of the Soviet Union. The term tends to minimize the intensity and courage expressed by the participants and is intrinsically both teleological (the inevitable sequence of winter-spring-summer-autumn) and a form of passivity (spring will come even if you refuse to take any action). In this regard, the word ‘spring’ shares something with the term ‘revolution,’ which is used in astronomy to refer to the movement of a celestial body around an axis in line with its orbit. The sequence of the seasons, as inevitable as it is, is coterminous to the cycles of celestial revolution; both are beyond the control of human agency. The conception of a revolution in European thinking as a sudden, radical change in the *status quo* derives from the effect of achieving a world upside-down, a *bouleversement* in the French version, which nonetheless is specular to the *status quo ante* and, as such, it is predictable. The idea of revolution as a radical change in history is in fact a modern concept, which dates back to the French and American revolutions of the nineteenth century. Before these events, revolutions did not indicate a radical transformation, but rather a rebellion or a *coup d’état*, which would not necessarily interrupt the course of history with a new beginning.^17^ Though it acquired a new dimension in modern times, the term ‘revolution’ referred to the idea of ‘restoration,’ to the reoccurrence of stages in life, forms of governments, good or bad fate. This means that originally the term referred to cycles of governments that periodically happen in human history whereas today it is used to refer to a structural change from the past. Along this trend, the term ‘revolution’ has been trivialized and made semantically void by its adoption, massively, in advertisement and media campaign as much as, *horresco referens*, by conservative politicians calling for change. A Google search for ‘revolution’ provides ample support for this claim;^18^ similarly, the slogan ‘the revolution is possible,’ used by Corrado Passera, Italian politician and banker with a career in US consulting firm McKinsey & Company—an emblem of corporate neoliberalism—is another disconcerting, example.^19^ When bankers adopt the language of revolt and revolution, it means that this political lexicon has been emptied of its politics.

The Middle East arguably has witnessed ‘uprisings,’ conceived as mobilization and oppositional moments at a popular level. Yet can we define these events as ‘revolutions,’ even when the people succeed in toppling oppressive rulers?^20^ Hannah Arendt argued that all revolutions are essentially expressions of taking action and starting something new, representing ‘the determination to act, [the] joy in action, the assurance of being able to change things by one’s own efforts.’^21^ If we limit our analysis to Arendt’s definition, the paradigm of ‘revolution’ would depict the events fairly well, as determination, joy and effort were key elements of the events in the Arab streets. Nonetheless, it is necessary to identify other layers of this revolutionary moment, if only for the sake of our theoretical framework.

The literature on revolutions preserves an overly historicized depiction of ‘revolutions’ and makes use of the past as a yardstick for the present. This methodology compares events in the Arab world with the cases of the 1989 Velvet Revolutions, the Iranian revolution of 1979 and the wave of European uprisings in 1848.^22^ While these revolutions had two main elements, the radical change to pre-existing political systems and the rise of charismatic leading figures, the Arab uprisings have not brought radical transformations, with the partial exception of Tunisia, and revolutionary leaders have not emerged in any of the countries. If leadership and radical structural change are essential to revolutions, it is inexact to use this analytical category for the events in the Arab world. There is a romance about revolutions as much as, in Lila Abu Lughod’s words, there is *romance of resistance.* Both impede at times the dissection of events in their political contexts and could be interpreted as categorical imposition. Yet one could add that they ‘can help detect historical shifts in configuration or methods of power.’^23^ Revolution, as much as resistance, remains a narrative frame, which is referenced by those whose lives occur in revolutionary times, both as participants, and as experiencing witnesses. The difference between these two remains in the fact that resistance is everywhere, perceived or unnoticed, but revolution remains epochal and defines a rupture—also in temporal terms. To paraphrase Rosa Luxemburg: *resistance is the means, revolution is its goal*.^24^

Others have interpreted the events as a fourth wave of democratization or a slowing down in the uptake of Samuel Huntington’s Third wave.^25^ The enthusiasm of democratization enthusiasts in applying democratization theories to every corner of the globe seems out of place and inadequate. In the case of the Arab revolts, a number of countries such as Egypt and Libya have toppled the ruler, but have re-produced respectively an enhanced military system and a militaristic chaos, the latter oxymoron we believe describes adequately post-Qadhdhaffi Libya. In both instances, popular participation and representation have been *de facto* denied, even in their most basic procedural forms, while Western forces have re-adopted the historical position that favors authoritarian stability over anything else.

Another expression that gained resonance in the Western semantic taxonomy is ‘Arab Awakening.’ The term was first used by George Antonius in his famous book *The Arab Awakening* (1938). The reutilization of this term carries two disputable assumptions: The first is that the events in the Arab world represented an unprecedented and non-predictable event, which occurred *ex abrupto*. The second is that they arose from a political, civic and emotional dimension that was not there previously, or that existed only latently. According to this reading, the revolts were the result of random occurrences that, by virtue of fortune, took root and spread like wildfire. Did Arabs awake from a long sleep and decide to pour into the streets in a moment of madness? Or, *a contrario*, did the West become suddenly aware of the transformations taking place in the MENA region when they erupted in 2011? Who actually was sleeping?

## Arabizing the Terminology of Revolt

‘Awakening’ traces its history back to the *naḥda* movement in the late-nineteenth century Arab-speaking world. It emerged soon after the Italian Risorgimento, with which it shares also the actual meaning of the word *naḥda,* and the proto-idea of a unitary nation. The term ‘revolution,’ in contrast, carries a variegated and pluralistic set of meanings. It is itself a site of struggle between diverging ways to interpret and, echoing Marx’s 11th thesis on Feuerbach, to change the world. In a modest bid at redrawing and re-assessing terminology, we shall pay heed to how revolution and its close associates (i.e., revolt, awakening and uprising) are translated in the Arabic language.^26^ For example, in modern standard Arabic the term *thawra* (which derives from the verb *thāra*: to revolt) is used to translate the term ‘revolution.’^27^ With a broad range of meanings, including insurrection, it originally indicated the idea of revolt rather than that of revolution.^28^ Indeed, it is another Arabic term, *inqilāb*, which best interprets the English word ‘revolution,’ with the meaning of ‘overturning,’ or the French ‘bouleversement.’ *Inqilāb* currently is used in Persian to translate the Western concept of revolution, both with its political and astronomic significance, whereas in Arabic, it unambiguously refers to the paradigm of *coup d’état*. One could dare say that the Western concept of revolution—being historical revolutions *invented* in the West^29^—does not effectively belong to the cultural milieu and political horizon of Arab societies, at least not in the way Western scholars have prefigured it.^30^

In the parlance of politicians in the Arab world, there have been several revolutions since the outset of the post-colonial era. The uprising against British colonial power in the summer of 1920 is recalled in Iraqi history books and throughout the Arab world as the ‘Iraqi revolution of the 1920’ (*thawrat al-‘ashrīn*). By all definitions, this event unwrapped along the lines of traditional anti-colonial uprising, yet the use of the term revolution gives it further legitimacy. The 1958 *coup d’état* that ousted the Hashemite monarchy in Iraq is also named officially as ‘the 1958 Revolution’ or the ‘July 14 Revolution.’ Similar considerations can be made for Egypt’s 1952 and Syria’s 1963 free officers’ *coups d’état*, or paradoxically to the 1980 ‘corrective’ purges led by Hafiz al-Asad, which went by the name of *al-thawrat al-taḥsisiya*, ‘the corrective revolution.’

Moreover, there is a subjective side, which derives from the voice of people who take part in popular movements of a revolutionary kind. As Rajesh Tandom and L. David Brown point out, Egyptians who toppled Mubarak call themselves revolutionaries, as do those who elected Muhammed Mursi and, paradoxically, those who supported Abdel Fattah el-Sisi’s coup.^31^ Hence, who are the revolutionaries and to what revolution is the reference? Again, revolutions are also narrative devices, to which political agents allocate certain phenomenological meanings in light of historical unsettledness. Thus, the term *thawra*, which means ‘revolt’ but is translated as, and often used, by Arabic speakers to refer to ‘revolution’—is a much abused one in the Arab world; lest one does not distinguish revolts from revolutions, heed should be paid to the use of terminology both in the local ecology and in the global analysis.

## Revolt, Revolution, Non-movement and Movement

We identify the characteristics of the events of post-2011 in the Arab world along several performance elements. Firstly, these events were popular manifestations of social and political disaffection, with no clear leadership, no structured political organizations (although political organizations took part in them), no unifying objective other than the *immediate*, ‘the people want the overthrow of the regime.’ One can define them as the combination of existing social movements, such as the array of civil and grassroots organizations (workers’ unions, women’s movements, students movements), and the non-movement of ordinary people, otherwise disengaged from active politics. Asef Bayat defines the non-movement as,

the collective actions of noncollective actors [which] embody shared practices of large numbers of ordinary people whose fragmented but similar activities trigger much social change, even though these practices are rarely guided by an ideology or recognizable leaderships and organizations.^32^

Bayat’s definition, which despite the author’s explication, is still remindful of Antonio Negri and Michael Hardt’s prefiguration of the *multitude* as a multifarious, manifold, multi-faceted entity made of individual subjectivities which bear immanent revolutionary power. Yet, Bayat’s anthropological sensitivity allows his definition to capture more accurately the potential of everyday ordinary practice against, or despite, the state authority. While the multitude of Negri and Hardt transmutes into an almost eschatological representation of humanity as a whole, Bayat, who is a scrupulous field observer and has a matchless feel for street politics, grounds his definition of non-movement in anthropology. More significantly for our analysis, though, the non-movement fits into our interpretation of the Arab Spring as a revolt, because the collective actions of the people (or the multitude) were concerned with the immediacy of the political action, never with the *status quo post*. In fact, the revolutionary momentum in Egypt, Syria and even Tunisia was mostly negative in its demands. It voiced popular resentment against what was not acceptable, injustice and the establishment. It was also the materialization of popular exasperation against the progressive worsening of socio-economic conditions and the widening gap between the elites (both political and economic) and the rest. It was, in the words of Walter Armbrust, ‘a revolution against neoliberalism’ in Egypt,^33^ but also in Tunisia (and other Mediterranean countries). It did not propose alterative models, but it aimed at destabilizing and dethroning previously established authorities, in furtherance of achieving dignity and economic security.

The Tunisian slogan *al-tashghil istihqaq ya ‘asabat al-sirqa* [Job is a right, you pack of thieves] accompanied with the universal prayer in Egypt *al-‘aysh*, *wal-horriyah wa ‘adalah ijtima’iyya* [Bread, Freedom and Social Justice) resonated all too familiar—also across time—to the Greek chant ‘Bread, education, liberty.’^34^ The climate of protest and contestation that characterized the 2000s, although often described by NGOs, international observers and liberal activists as non-political, were prelude to the spontaneous participation of the 2010s. Beinin captures this, ‘In both Egypt and Tunisia, most of the oppositional intelligentsia failed to appreciate that in an authoritarian regime, recurring mobilizations of large numbers of people seizing control of public space—workers, the unemployed, devotees of the “apolitical” preacher ‘Amr Khalid, or soccer fans—are inherently political.’^35^ In this instance lies also the common ground between the Arab revolts and the Western protest movement, as we will discuss later.

Let us dwell a little further on these points, by bringing what the scholarship of Middle Eastern Studies and, sadly, politics normatively have excluded. What can we identify as the horizon of political change among the multitude of people who took to the streets during the Arab uprisings? Political philosopher and anthropologist Furio Jesi has outlined the essential difference between revolution and revolt:

We use the word *revolt* [*rivolta*] to designate an insurrectional movement different from the revolution. The difference between revolt and revolution does not exist in the goals of one or the other; both *might* have the same goals: the seizing of power. That which distinguishes revolt from revolution is instead a different experience of time. If, according to the meaning of the two words, revolt is a sudden insurrectional outburst, which can be inserted into a strategic design, but which does not imply a strategy in the long term [*una strategia a lunga distanza*], and revolution is instead a strategic complex [*un complesso strategico*] of insurrectional movements […], one can say that revolt suspends the historical time and establishes a time when everything which happens has a value *per se*…the revolution instead is entirely and deliberately entrenched in the historical time.^36^

What does this distinction imply for our understanding of the transnational events collectively referred to as the ‘Arab Spring’? It shows that the two concepts of revolt and revolution, albeit different, exist in a symbiotic relationship: There can be no revolution without revolt, although the contrary is not generally true. Despite the several terms that we have scrutinized hitherto, it seems that, analytically, the ‘Arab Spring’ can be situated between two paradigms of political mobilization and change, that of ‘revolution’ and ‘revolt’. Their intrinsic difference lies in the conceptual timeframe of the movements that support them. Revolutions are struggles for the ‘after tomorrow’ and the ‘other yesterday,’ but revolts long for immediate results, for the ‘now’ and ‘tomorrow.’ They are the materialization of an overwhelming sentiment of impatience, which unfolds in the collective action of otherwise perceived passive communities and individuals. In this case, the revolts were against the marketization and profit-seeking authoritarian state (as embodied by the ruling elites, Gramsci’s *classe dirigente*).^37^ This struggle had been largely unnoticed for more than a decade, even though Egyptian, Greek and Tunisian workers, the unemployed and students had called for mass strikes, occupied public places and complained against degenerating precariousness and worsening economic conditions.^38^ For instance, Greek workers had called for a mass strike against unprecedented austerity cuts in May 2010, and five people had lost their lives on that occasion. A year later, in 2011, the Indignant movement in Greece occupied Synthagma Square, connecting its struggle with those in Tahrir Square.

Yet, if one considers the political developments in Egypt after the 2013 *coup d’état* by General Abdel-Fattah el-Sisi, it seems clear that the dynamics of the revolt have metamorphosed into a phenomenon, *à la* the theory of Antonio Gramsci, of *passive revolution*. This development conceals great analytical depth, as the events were marked by an active social upheaval, albeit of the disorganized type (the non-movement), which brought immediate results in the power contest. Indeed, the fall of Hosni Mubarak exemplified the success of the popular revolt, as much as it did in Tunisia with the ouster of Ben Ali. Yet, the small ruling elite which emerged from the unrest of post-Mubarak Egypt led to the creation of power and a new institutional framework much in line, both in the means of coercion and the structure, with the previous regime. New groups of people are now in power across the region, but the fundamental structures of government have remained largely unchanged. It is what Gramsci defines as a revolution without a revolution.^39^

Apart from buttressing our argument of the ‘Arab Spring’ as a moment of revolt, the concept of passive revolution also elucidates some of the trajectories of the current regime of power, for instance, in Egypt. Gramsci defines passive revolution as ‘a process whereby a social group comes to power without rupturing the social fabric (as in 1789 France) but rather by adapting to it and gradually modifying it.’^40^ The category of revolution holds ground inasmuch it is a narrative device among the people, yet it loses its momentum in face of counter-revolutionary dynamics. The revolution was aspired and narrated—and in that respect experienced as a manifested historical event, yet it did not materialize in substantial effects. Hierarchies were neither overturned nor abandoned.

As argued earlier, the Arab streets were shaken by disorganized arrays of ordinary people who united with formal groups, such as workers and students, who had maintained antagonism to the state over the preceding decade and now found synergies with a new multitude. The events situated in a historical time that was impacted by the economic crisis in Europe, exacerbating the multiple social crises already existing in the MENA. In other words, one could add that ‘the global crisis did not *cause* the revolts, but it was an essential ingredient of it.^41^ Arguably, the people, conceived as the collective of informal groups not directly engaged in institutional politics, led the revolutionary moment but failed to seize the symbols of power, in other words, to make space for their entry into a historical time.

The terminology provided by Jesi, whose analysis focused on the 1919 *Spartakus* revolt led by Rosa Luxemburg in Berlin, established a number of connections with our terminological analysis of the ‘Arab Spring.’ One confidently can state that what occurred in 2011 in several Arab countries has been a political revolt. This revolt, regardless of its outcome (which has been fragmentary and indiscernible), broke the historical time in which Arab countries had been imagined, both by local and global observers. If Arab societies were portrayed as passive—because inactive, and silent—because unheard, they could not be taken as the triggering model of revolt and opposition across the Global South and the West. Yet, in their demand for social justice, freedom and their opposition to the reduction of people to subjects of economic calculus, the identity of ‘Arab’ protesters resembled all too clearly that of protestors across the Mediterranean and the Atlantic.

## ‘Them’ *and* ‘Us’*:* Local Globalities or Global Localities?

Although the long-term outcome of the revolts in the Arab world is not clear, we can draw one conclusion: The results of the historical events defied all expectations and political-logical models. No linear transition to democratic governance occurred, including in Tunisia, but neither have the revolts, once and where sedated, generated a *status quo ante*. The euphoria of the first months evaporated into disillusion, expressed through a new terminology and new definitions, containing the sense of scepticism and failure felt. Observers began referring to the revolts as the ‘Arab Winter,’ and/or the ‘Islamist Winter,’ and detailing them with connotations that were much the opposite of their initial claims. Again, the paradigm of the impossibility of change and exceptionality of the Arab world had a comeback. The failure in understanding the events on the ground and those that pre-dated them reverberates in the narrative adopted by most of the international and local media, politicians and often academics, who seem to keep readopting the idea of ‘Arab exceptionalism.’ Joseph Massad refers to how Muslim and Arab countries were seen as incapable of embracing democracy, in the way Japanese, Indian and Latin American people have done, because of a presumed ‘*Islamic* failure to democratize.’^42^ It is sufficient to note how the same idea of an ‘Arab Awakening’ is a linguistic device to reiterate that same message, i.e., Arabs’ inability to produce democratic change.^43^

Western scholarship on the ‘Arab Spring’ has been largely deficient of an understanding of the phenomenon, not only because of a detachedness from the actual field of enquiry (the Arab streets and populace), but also because of its disconnectedness from political ferment in Europe and the United States.^44^ The latter conditions the interpretation of the Arab world and its politics greatly; it hampers the emergence of a coherent narrative of revolt between the events in the Arab world and the social protests in the capitals of the West. This is exemplified in the use of skilful linguistic and thematic choices. The most common and effective way has been that of emphasizing the diversity between ‘they’ (Arabs and Muslims) and ‘us’ (Western); and the adoption of tones at times pathetic, more appropriate to the description of exotic topics than everyday civil resistance.^45^ Yet, what is most extraordinary is the adoption of such a narrative not just by the West but also by local news outposts and elites.^46^ *De facto*, the West cocooned Arabs into a crystal casing, which paradoxically has been internalized by the same subjects. Edward Said had argued that Orientalism is not just an attitude of the West toward the Middle East, but also an attitude that is appropriated and interpreted by local elites and experts.^47^ The late Fouad Ajami is case in point of this self-Orientalization. An Arab observer with Western scholarly credentials and academic career, he exhibited the effect of Orientalism, as he recognized Arab exceptionalism as an actual phenomenon and attributed to it the causes for the intrinsic, regressive culture of Arab societies.^48^ Islam becomes, after the Orient, antonym of the West, or for that matter, secularity, whatever its actual content is. Crucially, Islam—and the Middle East consequently—operates as an internal category of Western politics, producing a perception of the West as liberal, tolerant and advanced.^49^ To put it more crudely, when Western protestors demonstrate, occupy squares, confront the police and challenge the political establishment as a whole (e.g., *Indignados* of Puerta del Sol or the Indignant Movement of Synthagma Square, etc.), they are framed as the discontent of the economic crisis and austerity, but when Arab protestors take to the streets they simply are mobilizing against dictatorship, for democratic government or *vice versa* barbaric bloodthirsty Islamists.

 However, there are similarities between the local (Arab) manifestation of dissent and the global (Western) movement against capitalism (and economic crisis-cum-austerity). They both appear as elements of one global localized community of protest, albeit differing in the immediate objectives for which they aspire. Both Arab and Western protestors share an underlying coherence of language of dissent, mobilization tactics, social ideals, and they also object to similar means of repression (with differing intensity). Understanding these shared elements has the benefit of framing the analysis of the ‘Arab Spring’ not as an exceptional and legendary event, but rather of shedding analytical insight on the events, the scope of Western political manifestations and, potentially, on their genealogy.

The emphasis on the new generations and their use of new social media technology is one way the Arab revolts prefigured events in Western cities. The Arab revolts have passed into history as the ‘Facebook, Twitter or YouTube revolutions,’ given the prominent role that these tools had in ensuring the burst of the revolts, promoting dissent, breaking the barrier of fear and coordinating the logistics of the upheavals.^50^ The binomial digital media and new generations tend to convey the impression that the Arab uprising has been caused by Western technology rather than the people in the region. In this regard, Tarak Barkawi asserted:

To listen to the hype about social networking websites and the Egyptian revolution, one would think it was Silicon Valley and not the Egyptian people who overthrew Mubarak.^51^

Differently from the codes of actions and revolutionary practices exemplified by the popular mobilization in the Global South (e.g., 1958 Cuba, 1979 Nicaragua, 1994 Chiapas), the tools of information technology and the internet transformed the fight into a *peaceful* one (read: non-violent, no bullets). Claims and demands turned around, in the West as in the Arab world, ‘real,’ ‘true,’ ‘radical’ or ‘participatory’ democracy; its fundamental, for how vague, objective remained political participation, representation and, as we discuss later, dignity among an ‘outraged’, ‘indignant’ population.^52^ In this, one also can identify the limits and, perhaps, weaknesses of both political events. In neither case, were the demands structured explicitly around a new model of power, of state, of political life. For however interesting and appealing ‘true democracy’ could be, it did not address through which means it would contain the intrusive, reactionary forces of capital, army and security. And as social media represented mostly demands of ‘freedom of expression,’ ‘free elections,’ and ‘democracy’ they also excluded the more contingent and, indeed, revolutionary program of working, popular classes, as emblematized by workers and the poor. For instance, groups of Egyptian workers after the fall of Mubarak, demanded, ‘If this revolution does not lead to the fair distribution of wealth it is not worth anything. Freedoms are not complete without social freedoms. The right to vote is naturally dependent on the right to a loaf of bread.’^53^ Yet, claims as these, largely were disregarded in the online communities, or were present only in its marginalia, similarly to the way European protests were portrayed as against the crisis, and not against the economic regime. Joel Beinin, once again, provides insight on a case from Tunisia:

Tunisian cyber-dissidence first emerged in 1998. But bloggers rarely discussed workers or protests in the interior regions. Many adopted an apolitical, ‘moderate’ stance. Lina Ben Mhenni (‘A Tunisian Girl’), one of Tunisia’s most acclaimed bloggers, was among the organizers of the May 22, 2010 protests in Tunis, Paris, Bonn, Montreal, and New York demanding an end to Internet censorship. They instructed participants not to bring ‘flags or music which could give a political or a religious meaning to the demonstration.’ Many Internet activists lived abroad. The *Debatunise* blog noted, ‘Cyber-dissident speech remains confined to a limited Tunisian reality responding to frustration of a particular social group. . . . There is no doubt that the members of this ‘club’ are the local or immigrant bourgeoisie.’^54^

Yet, the insolubility of popular demands through the social media sphere should not overshadow other meaningful effects. The use of social media is also significant because it shows how the Arab protestors preceded and offered a model for European and Western social movements. Firstly, Arab protestors made use of digital media in order to bypass the limits of mainstream, national media’s coverage of the events. By sending images and videos online, they also provided material models of organization to other protestors around the globe. One example above all is represented by the crucial spatial occupation of squares and parks, which first was performed in Tahrir Square and imitated, for example, in Madrid’s Puerta du Sol, Athens’s Synthagma, Barcelona’s Plaça Catalunya and Istanbul’s Gezi Park. This connected the visibility of the digital/virtual sphere of the new media with the material struggle for space in the squares. A leading Greek activist said, ‘in May 2011, the spirit of Egypt came via Spain to Greece.’^55^ He forgot to mention that more than Spain, it came from the digital connections that the activist community—as a communication *avant-garde* of the revolt—had with other movements across the Mediterranean. The Arabs not only reinterpreted and readopted the new media according to their needs, but also they struggled (by using them) to raise their voice above the Western tendency to adapt the news to their own views, so in a way the new media have become the means to bypass their national censorship and the one imposed by the West.

Although the connection between the Tahrir Square model and the mobilization in the European capitals is evident to the activist community, it has been misrepresented in the mainstream media. The new media tools, created mostly by Western companies and therefore an invention of the West, shorten distances and make the younger generations of the Arab revolt more similar to the European/American. They also enhance a class dimension, as poorer strata of the population have limited or no access to them and they are therefore *less* visible in the virtual space, as mentioned above in reference to Egyptian and Tunisian workers. Thus, the middle class of the Arab societies lies under the surface of the images, leaving the spotlight to young, often female, college and university students. As such, IT has had the potential to redeem the image of the Arab world as underdeveloped, dangerous, chaotic, violent, passive and in need of help (from the West). By doing so, the new technologies also discharge the new generations of the Arab world of the epithet of ‘terrorists.’ It gave credit to a reversed image of the Arabs, one that, ontologically, contrasted with the images and videos of al-Qaeda and ISIS-like groups. Hence, this type of reading seemed to assert that the new Arab generations are ‘modern’ in that they are IT-savvy, ‘peaceful’ in virtue of their civil disobedience and non-armed resistance; ultimately, they were at odds with Islamist fundamentalists. Some observers even interpreted the ideology of the Arab youth as inspired by the ideas of non-violence theorist Gene Sharp, of whom most Arabs probably had never heard.^56^ The paradox, after 2013, comes with the emergence of ISIS and their telegenic and cyber-savvy use of social media, which are deployed almost exclusively to foment sectarian hatred, celebration of violence and spread of terror. The young, defiant Arabs return to the background as the revolts acquiesce on the surface, nonetheless, of digital screens.

Furthermore, the perception of Arab and Islamic identity with terrorist organizations in European discourse is still not settled.^57^ A report by the EUROPOL about terrorism in Europe pointed out that in 2013 there were 152 ‘terrorist attacks’ in European states, of which only two were connected to religious (read Islamic) extremism. The remaining bulk of the attacks were connected to separatist and anti-systemic organizations, often belonging to far-right, xenophobic cells. Yet, national news outlets have been referring to the danger of Islamist groups operating on European soil, well ahead of the events in Paris during 2015; very little reference is made to right-wing groups, some of which also are represented in national parliaments. Indeed, the Europol report highlighted the need for increasing surveillance of individuals across the EU, implying stricter control on personal movement, political and religious activity, all of which have been materializing after the coordinated attacks by ISIS in Paris in November 2015. All of this occurs in the context of tighter measures on the phenomenon of refugees and the covering up of Saudi-Turkish relations with Islamic State fighters.^58^ Besides buttressing and consolidating the perception of an Islamic threat in Europe, it seems that the measures proposed by national governments have other, more political, objectives. To which extent, one could contend, the security measures applied by France, the UK and other European countries are aimed at curbing Islamic terrorism and not at increasing the state’s potential of preventing social mobilization in times of economic crisis?

In fact, there is resemblance between the post-revolutionary measures in Egypt, Libya and other Arab countries and the security measures taken by the Greek, Spanish and UK governments in the wake of popular protest. The emergency laws by Egypt’s government-*cum*-army, both nationwide and in the Sinai, embody this post-revolutionary governmentality. In November 2013, the Egyptian interim president imposed hefty fines on any public gathering of more than 10 persons, without previous governmental approval.^59^ Similarly, in the wake of the mass movement of the *Indignados*, the right-wing government in Spain pushed forward legislation, which would ‘elevate passive resistance to a criminal offense, including blocking the entrance to public buildings and sit-ins.’^60^ The law applies fines of up to $600,000 Euros for unauthorized demonstrations in front of the Parliament and up to $30,000 Euros for insulting the police.^61^ More recently, the UK parliament has passed a law undermining the capacity of civil society, and especially trade unions, to put pressure on the government. The UK government thus is able to impose fines on unions for not having a completely accurate membership list and impose restrictions to civil society campaigning ahead of elections, through a combination of bureaucratic maze and heavy fines.^62^ The list of measures along the lines outlined above is long and we shall not dwell longer on it to demonstrate its relevance.

There are other shared elements in the unwrapping of Arab and Western protest movements. The horizontalism of Arab protesters entered in a dialogic and mutual relation with the Western activist communities, despite the little recognition that it has had hitherto. As Paul Mason’s interviews with key participants confirm, horizontalism was the main feature of the Arab protestors’ *modus operandi*.^63^ Informal, participatory and direct democracy groups operated and communicated with each other across the Mediterranean and the Atlantic. Even in the case of Syria, since the beginning of the uprising, local committees and councils began to operate out of the blue, with a broad network of participants and with no aid or support from external entities. Conceptualized by the thought of Syrian anarchist Omar ‘Aziz, who had died before Western media could pay attention to him, the local councils were forms of autonomous, non-hierarchical, self-governed organizations based on the principle of cooperation, mutual aid and psychological support.^64^ The local committees embrace all segments of the society; they are structured horizontally, deal with the provision of basic services, coordinate with the local councils and with armed resistance groups, and allow for local management of security concerns.^65^

Indeed, the self-organization of protestors and civic activists, whether in Tahrir Square, in Aleppo’s war-torn neighbourhoods, or Zuccotti Park in New York, had similar opposition tactics. David Graeber, anthropologist and long-life activist, shows in his book, *The Democracy Project*, how protestors in Egypt, Barcelona, Athens and New York had channels of communication about everyday matters of struggle, such as how to defy police surveillance, and how to self-organize a horizontal and deliberative meeting among numerous protestors. They also shared their views about the strategy adopted by the police and the security apparatuses to deter them.^66^ To provide a rather facetious yet symbolic image of the inter-connectedness of local Arab protests and global anti-capitalist protest, Graeber brings in the role of pizza express. ‘Inspired by the example of the Egyptian labor unions that had sent pizzas to fellow union activists occupying Wisconsin’s statehouse some months before,’ Graeber writes, ‘hundreds of people across North America and beyond reached for their credit cards and began phoning in orders for pizzas.’^67^ The pizza connection is a more profound token of global solidarity among subaltern groups, of the variegated and unclassifiable type. It also unveils a common thread among the local protesters in the Arab streets, which, as we argued above, did not have politico-ideological schemata, but manifested their will to dignity [*karamah*] and justice [*‘adalah*], and the global protesters of the Occupy movements. After all, *Indignado* is the Spanish word for ‘outraged’, which, derives from the word ‘dignidad’, ‘dignity’, *karamah*. Dignity might turn out to be a catchall for the aspirations, demands and idea(l)s of protestors across the Mediterranean and the Atlantic and, as such, might ‘obscure its economic and social aspects and the aspirations of its initiators.’^68^ In this respect, ‘dignity’ is unifying, yet homogenizing; it brings the poor and the workers on the side of students and intellectuals; it bridges the epistemic divide between the Arabs and the rest; it refers to a human gasp which is undeniable and yet it declines, at times, the imperative to materialize dignity, a step that necessarily prefigures contradictions. What does this ‘dignity’ ultimately signify? The concept seems to unfold common contradictions, aspirations and struggles in the West as in the East. Hence, how to integrate these phenomena across social, cultural and economic localities, into a unique, yet dynamic, global scholarship?

## Conclusion*:* A Scholarship of Silence

In the past two decades, the social sciences have been overwhelmingly occupied with evaluating to which extent theories of democratic rule have been adopted and respected across the globe, in particular the non-Western world. One implication of these, writes authoritatively the political scientist Paul Brass, ‘is that we have been unaware of the kind of society we live in, […], of the confined, restricted, and ultimately inconsequential character of our participation in political life as citizens.’^69^ One could add that we have been also unaware of our political life as academics, unaware of the ‘passive’ actions of political subjects across the Mediterranean region, as much as we have been deaf to the silent voices of all those who expressed in words, arts, acts and virtual signs a semantic of revolt against state power and the *status quo*. This changing consciousness, which preluded to the outburst of popular uprisings in the Middle East, did not go hand in hand with the transformed hermeneutics of the region, given the fact that the majority of the academic community of Middle Eastern studies operated in their everyday scholarly existence as consultants, experts of high politics and disengaged intellectuals. This epistemic community could not possibly capture the region’s new perceptions and practices while it was unearthing new models of grassroots politics. The objectives of the epistemic community were narrowly focused on evaluating, ‘calculating’ and schematizing old/new political alliances, electoral procedures, transparent administrative mechanisms to coherent and compatible (to the West’s capitalist regime) laws and legislations. To an extent, this corroborates Žižek’s analysis of public life and politics as based on the idea of ‘economy as ideology’, globally;^70^ one could add that the annex to this knowledge/power has become *security as ideology*.

To this scholarship, we counterpoise and encourage for what Asef Bayat defines as a *scholarship of silence*.^71^ Through the hermeneutics of the passive, silent and revolutionary existences of ordinary people, of established and underground institutions, of cultural, economic as well as scientific, technological phenomena,^72^ today’s epistemic community can discern what cannot be taxominized in questionnaires, numerical schemata, statistical cases and elite interviews. As the practical boundaries of this essay did not allow a thorough exemplification of this kind of scholarship, our key objective has been to demonstrate, through both theoretical and observational instances, its validity. This scholarship reads the silences in the news; it attempts to identify and to interpret the disorderly monotony of the streets and of contentious existences in spite of the state and of Western misinterpretations of the rest of the world.^73^ Its undertaking is, alas!, piecemeal, but its potential endless.

## Acknowledgements

The authors would like to thank the two anonymous reviewers for their thought-provoking and insightful comments on the first version of this article, which originally was presented as a paper at the XII Annual Conference of the Italian Society for Middle Eastern Studies, held at the University of Venice in January 2015.

## Disclosure statement

No potential conflict of interest was reported by the authors.

## Footnotes

^*1*^ Errico Malatesta, inscription on a monument in Pozzuoli, Italy.

^2^ Due to the lack of precise and consistent terminology to refer to the MENA region, this article uses the expressions ‘Arab world,’ ‘Middle East’ and ‘region’ interchangeably, with no intention of excluding non-Arab populations.

^3^ See B. Lewis (1982) The Question of Orientalism, *New York Review of Books*, June 24, 1982.

^4^ B. J. Brownlee (2015) The Revolution ‘from Below’ and Its Misinterpretations ‘from Above, The Case of Syria’s Neglected Civil Society, *Syria Studies*, 7(1).

^5^ L. Wedeen (1999) *Ambiguities of Domination: Politics, Rhetoric, and Symbols in Contemporary Syria* (Chicago: University of Chicago Press); and A. Sottimano (2008) Ideology and Discourse in the Era of Ba’thist Reforms: Towards an Analysis of Authoritarian Governmentality, in: A. Sottimano & S. Kjetil (eds) *Syria Studies*: *Changing regime discourse and reform in Syria* (Fife: Centre for Syrian Studies), 1–55.

^6^ M. Shihade, C. F. Fominaya & L. Cox (2012) The Season of Revolution: The Arab Spring and European Mobilizations, *Interface*, 4(1), pp. 1–16.

^7^ G. Deleuze & F. Guattari (1988) *A Thousand Plateaus: Capitalism and Schizophrenia* (London: Bloomsbury Publishing), p. 7.

^8^ A. Bayat (2014) After the Arab Spring, in *BRISMES ‘The Middle East in Global Perspective: Interactions in Time and Space’* (Brighton, UK: University of Sussex).

^9^ S. P. Huntington (1993) *The Third Wave: Democratization in the Late Twentieth Century* (Norman: University of Oklahoma Press), p. 4.

^10^ G. Gause III (2011) Why Middle East Studies Missed the Arab Spring: The Myth of Authoritarian Stability, *Foreign Affairs*, 90(4, July–Aug.).

^11^ Brownlee, The Revolution ‘from Below’.

^12^ See D. Jolly (2013) Death Toll in Syrian Civil War near 93,000, UN Says, *The New York Times*, June 13, 2013. Available at http://www.nytimes.com/2013/06/14/world/middleeast/un-syria-death-toll.html?_r=0, accessed March 3, 2016.

^13^ M. Hudson (2011) Awakening, Cataclysm, or Just a Series of Events? Reflections on the Current Wave of Protest in the Arab World, *Jadaliyya*, May 16, 2011. Available at: http://www.jadaliyya.com/pages/index/1601/awakening-cataclysm-or-just-a-series-of-events-ref, accessed March 3, 2016; and B. Haddad, R. Bsheer & Z. Abu-Rish (eds) (2012) *The Dawn of the Arab Uprisings: End of an Old Order?* (London: Pluto Press. *Cf*. A. Alexander (2012) Digital Generation, in: Z. Sardar & R. Yassin-Kassab, *Critical Muslim 1: The Arabs Are Alive* (London: Hurst & Co).

^14^ F. Halliday (1999) *Revolution and World Politics: The Rise and Fall of the Sixth Great Power* (Durham, NC: Duke University Press), p. 25.

^15^ W. A. Gamson & G. Wolfsfeld (1993) Movements and Media as Interacting Systems, *The Annals of the American Academy of Political and Social Science*, pp. 114–125.

^16^ W. Benjamin (1996) The Metaphysics of Youth, *Walter Benjamin: Selected Writings* 1 (London: Harvard University Press**)**.

^17^ H. Arendt (1972) *Crises of the Republic: Lying in Politics; Civil Disobedience; on Violence; Thoughts on Politics and Revolution* (London: Houghton Mifflin Harcourt), p. 15.

^18^ First results of a Google search include: ‘Revolution Vodka Bar’, a US TV series ‘Revolution’; and a list of other companies and articles which refer to ads and marketing,

^19^ See http://www.italiaunica.it/, accessed on November 27, 2015.

^20^ G. Achcar (2013) *People Want: A Radical Exploration of the Arab Uprising* (London: University of California Press), pp. 13–15.

^21^ Arendt, *Crises of the Republic*, p. 206.

^22^ P. Mason (2013) *Why It’s Still Kicking Off Everywhere: The New Global Revolutions* (London: Verso Books).

^23^ L. Abu‐Lughod (1990) The Romance of Resistance: Tracing Transformations of Power through Bedouin Women, *American Ethnologist*, 17(1), p. 48.

^24^ R. Luxemburg (1966) *Social Reform or Revolution* (London: Merlin Press).

^25^ L. Diamond (2011) A Fourth Wave or False Start, *Foreign Affairs*, 90(3), p. 22.

^26^ Among the Arabic terms that gained resonance in the Western imaginary, *intifada* is probably unique. Translated in English as ‘uprising,’ it usually refers to the context of Palestinian mobilization against the Israeli occupation, with the objective of ‘shaking off’ (*intifadà*) the occupation. Because of the historical use of the term, Arab observers have avoided using this term in the context of the ‘Arab Spring’.

^27^ Emblematic of the orientalizing school, in this case, is Bernard Lewis, who explained that the Arabic word for revolution (*thawra*) derived from the rising up (of a camel) and, therefore (sic!), ‘revolt’. See J. A. Massad (2015) *Islam in Liberalism* (London: University of Chicago Press), p. 10.

^28^ The term was fairly translated into English when referring to the Great Arab Revolt of 1916–18, the 1920 Revolt in Iraq, the Great Syrian Revolt of 1925, and the Great Palestinian Revolt of 1936. See Achcar, *People Want*, pp. 14–15.

^29^ Together with *nations, societies and states*. See respectively E. Hobsbawm and T. Ranger (2012) *The Invention of Tradition* (Cambridge: Cambridge University Press); B. Anderson (2006) *Imagined Communities: Reflections on the Origin and Spread of Nationalism* (London: Verso Books).

^30^ On the delusions of etymology see F. Halliday (1993) ‘Orientalism’ and its Critics, *British Journal of Middle Eastern Studies*, 20(2), pp. 145–163.

^31^ R. Tandon & L. D. Brown (2013) Civil Societies at Crossroads: Lessons and Implications, *Development in Practice*, 23(5 –6), pp. 794–796.

^32^ A. Bayat (2013) *Life as Politics: How Ordinary People Change the Middle East* (Amsterdam: Amsterdam University Press), p. 14.

^33^ W. Armbrust (2011)The Revolution against Neoliberalism, *Jadalliya,* February 23, 2011. Available at: http://www.jadaliyya.com/pages/index/717/the-revolution-against-neoliberalism-, accessed March 22, 2016.

^34^ J. Beinin (2015) *Workers and Thieves: Labor Movements and Popular Uprisings in Tunisia and Egypt* (Stanford: Stanford University Press); and M. Simiti (2014) Rage and Protest: The Case of the Greek Indignant Movement (London: LSE GreeSE Papers).

^35^ Beinin, *Workers and Thieves*, Chapter 4, pp. 97–134.

^36^ F. Jesi (2000) *Spartakus: Simbologia Della Rivolta* (Torino: Bollati Boringhieri), p. 19.

^37^ Once again, translation are problematic, as the English translation of this term is ‘ruling class’ while Gramsci intended the hegemonic power which also to set the direction of politics, inherent in the *classe dirigente*.

^38^ Beinin indicates that the number of actions among these groups increased from *ca*. 200 in 2002–2003 to *ca*. 600 (plus *ca*.700 alternative actions) in 2006, 2007 and 2008; Beinin, *Workers and Thieves*, pp. 61–96.

^39^ See A. Gramsci (1967) *Scritti Politici* 1 (Roma: Editori Riuniti).

^40^ D. Forgacs (2000) *The Antonio Gramsci Reader* (New York: Lawrence and Wishart), p. 247.

^41^ A. Hanieh (2013) *Lineages of Revolt: Issues of Contemporary Capitalism in the Middle East* (Chicago: Haymarket Books), p. 162.

^42^ Massad, *Islam in Liberalism*, p. 32.

^43^ Brownlee, The Revolution ‘from Below.’

^44^ In one of the most enlightened contribution to social movement scholarship, there is only a vague allusion that there might be correlation between the waves of protest in Western capitals and the ‘Arab Spring,’ see D. della Porta & A. Mattoni (2014) *Spreading Protests: Social Movements in Times of Crisis* (Colchester: ECPR Press).

^45^ R. El-Mahdi (2011) Orientalising the Egyptian Uprising, *Jadaliyya*, April. Available at: http://www.jadaliyya.com/pages/index/1214/orientalising-the-egyptian-uprising, accessed March 22, 2016).

^46^ Brownlee, The Revolution ‘from Below.’

^47^ See B. Haddad (2013) Ajamindustry, in: A. Iskander & B. Haddad, *Mediating the Arab Uprisings* (Washington, DC: Tadween Publishing).

^48^ F. Ajami (2012) *The Syrian Rebellion* (Stanford: Hoover Press).

^49^ Massad, *Islam in Liberalism*, Introduction.

^50^ Brownlee, The Revolution ‘from Below.’

^51^ M. Ben Moussa (2013) From Arab Street to Social Movement: Re-Theorizing Collective Action and the Role of Social Media in the Arab Spring, in: M. Taki & L. Coretti (eds) The Role of Social Media in Arab Uprisings – Past and Present, *Westminster Papers in Communication and Culture*, 5(2), p. 47.

^52^ D. Porta & Mattoni, The Transnational Dimension. For ‘rage’ and ‘outrage’, *cf*. B. Moore (1978) *Injustice: The Social Bases of Obedience and Revolt* (New York: Sharpe White Plains).

^53^ Beinin, *Workers and Thieves*, p. 100.

^54^ Ibid.

^55^
*Solidarity Net* (2012) Greek activist: ‘The Movement has Brought Down two Governments’, June 14, 2012. Available at http://www.solidarity.net.au/greece/greek-activist-%E2%80%98the-movement-has-brought-down-twogovernments%E2%80%99/; accessed March 22, 2016.

^56^ A. Abu Khalil (2011) How to Start a Revolution: Or the Delusions of Gene Sharp, *Al-Akhbar English*, December 2, 2011. Available at http://english.al-akhbar.com/node/2169, accessed March 22, 2016.

^57^
*Aljazeera English* (ed.) (2015) ‘Extremism’: Perception vs Reality?, *Inside Story*.

^58^ K. Daoud (2015) Saudi Arabia, an ISIS That Has Made It, *The New York Times*, November 20, 2015.

^59^
*The Telegraph* (2013) Egypt’s Interim President Issues Strict Law Restricting Protests, November 25, 2013.

^60^ Shihade, Fominaya & Cox, The Season of Revolution, p. 8.

^61^ A. Kassam (2014), Spanish Government Drafts Strict Anti-Protest Laws, *The Guardian*, November 21, 2014.

^62^ R. Mason (2014) Lobbying Bill Passes through Houses of Lords, *The Guardian*, January 28, 2014.

^63^ Mason, *Why It’s Still Kicking Off Everywhere*, pp. 55–56.

^64^ B. Hassan (2013) *Random Shelling Blog*, February 20, 2013. Available at: http://budourhassan.wordpress.com/2013/02/20/omar-aziz/.

^65^ Brownlee, The Revolution ‘from Below,’ pp. 46–48.

^66^ D. Graeber (2013) *The Democracy Project: A History, a Crisis, a Movement* (New York: Spiegel & Grau), pp. 18–19, 106.

^67^ Ibid., p. 178.

^68^ Beinin, *Workers and Thieves*.

^69^ P. R. Brass (2000) Foucault Steals Political Science, *Annual Review of Political Science* 3(1), p. 311.

^70^ S. Žižek (2010) A Permanent Economic Emergency, *New Left Review* 64(1).

^71^ Bayat, After the Arab Spring.

^72^ A case in point of a scientific phenomenon intertwining with historical change is T. Mitchell (2002) *Rule of Experts: Egypt, Techno-Politics, Modernity* (London: University of California Press).

^73^
*Cf*. I. Volkmer (2012) *The Handbook of Global Media Research* (Chichester, UK: John Wiley & Sons), p. 205.
